# Editorial: Sex differences in cognition and psychological outcomes in chronic diseases

**DOI:** 10.3389/fpsyt.2024.1414613

**Published:** 2024-07-15

**Authors:** Manuela Altieri, María Dolores Roldán-Tapia, Gabriella Santangelo

**Affiliations:** ^1^ I Division of Neurology, Department of Advanced Medical and Surgical Sciences, University of Campania “Luigi Vanvitelli”, Naples, Italy; ^2^ Department of Psychology, University of Almería, Almería, Spain; ^3^ Department of Psychology, University of Campania “Luigi Vanvitelli”, Caserta, Italy

**Keywords:** sex, biological sex, sex differences, cognition, chronic disease, neurological disease, psychiatry

Biological sex is a multifaceted construct based on several anatomical, physiological, genetic, and hormonal variables (1). Differences between the sexes have been noted in several areas, including the structure and functionality of the brain; cognitive abilities; prevalence, disease course, and symptoms in neurological/psychiatric diseases; and psychological traits.

Sexual dimorphism in human brains is widely recognized in scientific literature: for example, brain volume is 10% larger in men; on the other hand, after correcting for intracranial volume effect, women seem to have a higher percentage of grey matter, although there are studies that do not confirm these findings (2, 3). Studies on specific brain regions found that frontal and medial paralimbic regions and Broca’s area (Broadmann’s area 44 and 45) were larger in women, whereas men exhibited a higher volume in the hypothalamus, the amygdala, and the angular gyrus (4). Moreover, analysis of the human connectome revealed sex-specific modalities of organization and integration of brain networks: within-hemispheric connectivity was higher in men, whereas between-hemispheric connectivity was enhanced in women. This result was found only in supratentorial regions, whereas in cerebellar regions the authors found an inversion of the abovementioned results (5). Taken together, these differences have been identified as the neural substrate that would explain sex variance in cognitive performance. Although it is now known that some imbalance in terms of performance on specific tasks may be ascribed to non-biological factors (i.e., gender norms and access to education), several studies found that men tend to outperform women in spatial tasks, whereas women show better performance on verbal tasks (6).

Biological sex may also be considered a risk factor for several chronic pathologies, such as neurological and psychiatric diseases, with a higher susceptibility to neurodevelopmental disorders and Parkinson’s disease in men, and a higher susceptibility to Alzheimer’s disease, Multiple Sclerosis, and depressive and anxiety disorders in women (7). Sex seems to also influence disease course: in Multiple Sclerosis and Parkinson’s disease the prognosis seems to be poorer in men and women, respectably (8, 9).

Given the importance of biological sex as a variable that needs to be addressed in health studies, this Research Topic aimed to explore the possible role of biological sex in several chronic diseases. The studies included in this Research Topic were thoroughly peer-reviewed and provided excellent insight into sex discrepancies in psychological and clinical outcomes.

The study *Longitudinal progression of choroid plexus enlargement is associated with female sex, cognitive decline and ApoE E4 homozygote status* (Novakova Martinkova et al.) explored the possible relationship between longitudinal changes in choroid plexus volume, biological sex, and cognitive deficits. Six hundred and thirteen subjects were evaluated, and the authors found that the rate of increase of choroid plexus was more than doubled in females compared to males; moreover, only the group of convertors (who developed dementia in follow-up evaluations) exhibited an increase over time of choroid plexus volume.

Unlu et al. in *Sex difference in alcohol withdrawal syndrome: a scoping review of clinical studies*, performed a scoping review aimed at comparing symptoms, clinical course, complications, and treatment differences between men and women in alcohol withdrawal syndrome (AWS). The review - including 35 observational studies - revealed that the clinical manifestation of AWS differed between sexes: men exhibited more complications of alcohol withdrawal syndrome (i.e., delirium tremens and alcohol withdrawal seizures) and mortality rate compared to women; moreover, they were more likely to receive benzodiazepine treatment.

In the cross-sectional study *Gender differences in the association between anxiety symptoms and thyroid hormones in young patients with first-episode and drug naïve major depressive disorder* (Zhao et al.), the authors found sex differences related to clinical variables and anxiety symptoms in patients with major depressive disorders; in more detail, age of onset was lower in men compared to women. On the other hand, levels of anti-thyroglobulin antibody were associated with anxiety symptoms only in the women group.

Levels of anxiety in people with immune-mediated inflammatory diseases were investigated in *Sex differences evident in elevated anxiety symptoms in multiple sclerosis, inflammatory bowel disease, and rheumatoid arthritis* (Joyees et al.). This study, including six hundred and fifty-six participants, provided evidence that women were more likely to experience elevated levels of anxiety compared to men; on the other hand, lower income was associated with higher anxiety only in the men’s group. Moreover, men were more likely to endorse the item regarding restlessness on the Generalized Anxiety Disorders-7 scale.

Finally, Hendriks et al. in *Gender discrepancies and differences in motor and non-motor symptoms, cognition, and psychological outcomes in the treatment of Parkinson’s disease with subthalamic deep brain stimulation* explored the potential effect of deep brain stimulation of the subthalamic nucleus (STN-DBS) for the treatment of motor and non-motor symptoms in advanced Parkinson’s Disease. This systematic review of 41 studies found that motor and non-motor symptoms responded differently to STN-DBS in men and women: men were more likely to have an improvement of bradykinesia, impulsivity, voice quality, fat-free mass, pain, and restless leg syndrome compared to women. On the other hand, an improvement in cognitive performance and urinary symptoms seemed to be more pronounced in women.

In conclusion, this Research Topic aimed to further investigate the possible sex differences in psychological and clinical factors in people with chronic disease. The editors would like to thank all authors who provided their invaluable contributions to this topic. Future studies are indeed needed to further explore this topic to define clinical protocols for tailored therapies considering the sex of the patient.

## Author contributions

MA: Conceptualization, Supervision, Writing – original draft, Writing – review & editing. MR: Writing – original draft, Writing – review & editing. GS: Writing – original draft, Writing – review & editing.
